# Editorial: Evolution of short genomic regions: discoveries, methods, and challenges

**DOI:** 10.3389/fbinf.2026.1821711

**Published:** 2026-03-18

**Authors:** Helen Piontkivska, Fabia Ursula Battistuzzi, Tzu-Chiao Chao, Nicole Hansmeier

**Affiliations:** 1 Department of Biological Sciences, Kent State University, Kent, OH, United States; 2 Department of Biological Sciences, Oakland University, Rochester, MI, United States; 3 Institute for Data Science, Oakland University, Rochester, MI, United States; 4 Department of Biology, University of Regina, Regina, SK, Canada; 5 Luther College at the University of Regina, Regina, SK, Canada

**Keywords:** comparative genomics, genome complexity, k-mer analysis, low-complexity regions (LCRs), network-based clustering, repeat-aware transcriptomics, sequence entropy, threshold-based detection

Genome complexity is an umbrella term that reflects the existence of multiple structural levels of genome organization, from compositional biases and differential gene expression to higher-order genome architectures. This includes whole genome duplications, pan-genomes, and 3D-folding, as well as how these features are regulated to become actionable instructions for a cell. A central challenge in studying genome complexity is therefore methodological: how can complexity be robustly defined, detected, and quantified in practice, particularly across divergent taxonomic groups. Existing approaches vary widely, with some emphasizing large-scale genome architecture and others focusing on gene structure, sequence composition, or functional output. The second, closely related challenge, concerns functional interpretation: how do we predict and validate functional implications of various aspects of genomic complexity. The third challenge is driven by the selected target, whether it be coding or non-coding sequences within a genome or a comparative approach across taxonomic levels. Given the wide and inconsistent use of the term “complexity”, it remains unclear how different operational definitions relate to biological function. As a result, methodological choices such as thresholds, descriptors, and even terminology, strongly influence which features are identified as complex and how their biological relevance is inferred.

This collection showcases on one hand the diversity of conceptual and methodological frameworks currently used to identify, name, and interpret low-complexity regions (LCRs) and related genomic features, but also illustrates the need for a consensus framework as authors rely on distinct, context-specific definitions of complexity, each revealing different biological insights. The common thread among this collection is the identification, across domains of life and widely different methodological approaches, of a functional role for low complexity regions, highlighting the need to develop new approaches to better understand this poorly studied feature of genomes.

Saravanan et al. adopt a threshold-based definition of low complexity, identifying LCRs across three closely related enterobacteria using the SEG algorithm with default parameters. Their results show that LCRs were consistently enriched in core and orthologous genes rather than in accessory or paralogous ones. This enrichment is particularly pronounced in genes involved in cell cycle control and defense, suggesting that LCRs may play conserved, albeit currently unknown, functional roles, rather than acting primarily as agents of evolutionary plasticity. Notably, this conclusion is inseparable from the specific threshold-based framework used to define LCRs.

On the other hand, Vaglietti et al. use the amino acid composition and two scores of per-residue sequence complexity reflecting the local degree of sequence simplicity (SIM) and repetitiveness (REP). Applying these metrics to a vertebrate gene family of translational regulators, namely, cytoplasmic polyadenylation element-binding proteins (CPEBs), the authors show a multitude of paralog-specific evolutionary trends in composition- and function-related parameters driven by divergence in LCRs and homopolymeric amino acid repeats (AARs). These regions are thought to mediate liquid-liquid phase separation (LLPS) and prion-like behavior of CPEBs.

Weaver et al. extend the concept of complexity to a population-level and network-based scale, describing a novel threshold-based approach in the context of viral transmission networks for molecular surveillance. Using solely genetic sequence data, AUTO-TUNE overcomes a frequent limitation of missing metadata and allows identification of clusters of related viral sequences across adaptively tuned thresholds. This approach is predicted to facilitate public health interventions that are appropriate to each specific outbreak. Here, complexity is not a sequence property *per se*, but an emergent feature of population structure, illustrating how threshold choice critically determines biologically and epidemiologically relevant inferences.

Sciaraffa et al. address yet another dimension of genomic complexity by focusing on non-coding repetitive sequences and their contribution to human disease phenotype. Using repeat-aware, transcript-centric analyses, they show that in a small cohort study the expression of AmnSINE1, a member of amniota-specific SINE family, is reduced in individuals with autism spectrum disorder and suggest a role for AmnSINE1 transcripts in miRNA-mediated neurogenesis. Their study underscores how non-coding repetitive elements represent a functional layer of complexity that is invisible to gene-centric pipelines.

Yet, another application of complexity to population-level inferences is described in (Ulyanov et al.). Their study combines k-mer based analysis of whole genome sequences and analysis of mitochondrial genomes to evaluate genetic variation in populations of critically endangered antelope *Saiga tatarica tatarica*. The results show heterogeneous distribution of variants across both nuclear and mitochondrial genomes and offer potentially actionable insights into genetic diversity of this species for future conservation efforts.

Overall, this collection highlights the current need for better understanding of architecture and function of low complexity regions, both within coding and non-coding contexts, across the entire Tree of Life. Current approaches differ not only in thresholds and metrics, but also in terminology and underlying assumptions, making cross-study comparisons difficult. Future studies should consider comparing how different thresholds for defining LCRs and other complexity-relevant genomic features affect functionally relevant inferences and explore whether common principles can be established across both coding and non-coding contexts and throughout the Tree of Life. Moreover, as illustrated by the current studies, the existing measures of complexity vary in how they are captured across levels of organization, from proteins to animal populations, underscoring the need to develop a common definition.

